# Emerging biomarkers and frontier therapies: unveiling the role of endothelial dysfunction in cerebral small vessel disease

**DOI:** 10.3389/fneur.2025.1615883

**Published:** 2025-07-11

**Authors:** Kedi Sun, Hui Liu

**Affiliations:** Department of Neurology, Kaifeng Central Hospital, Kaifeng, China

**Keywords:** endothelial dysfunction, cerebral small vessel disease (CSVD), blood–brain barrier, emerging biomarkers, endothelial cells (ECs)

## Abstract

Cerebral small vessel disease (cSVD), a major contributor to stroke, cognitive decline, and vascular dementia, accounts for around 25% of ischemic strokes and significantly impacts age-related neurological disability. Despite its clinical significance, the underlying mechanisms of cSVD remain incompletely understood, and therapeutic options are limited. Mounting evidence has pinpointed endothelial dysfunction as a central driver in cSVD pathogenesis, which disrupts blood–brain barrier (BBB) integrity, impairs cerebral blood flow autoregulation, and promotes neuroinflammation. The vascular endothelium, serving as a dynamic interface between blood and brain parenchyma, plays a crucial role in maintaining vascular homeostasis through functions like nitric oxide (NO)-mediated vasodilation, anti-thrombotic signaling, and immune regulation. In cSVD, chronic endothelial injury triggered by factors such as hypertension, oxidative stress, or genetic predisposition leads to microvascular rarefaction, pericyte loss, and gliosis, ultimately resulting in characteristic manifestations like white matter hyperintensities, lacunar infarcts, and cerebral microbleeds. Our review stands out by comprehensively integrating the latest research on emerging biomarkers and frontier therapeutic strategies specifically related to the cSVD-endothelium interplay. Recent breakthroughs in biomarker discovery, including novel circulating endothelial microparticles subtypes and advanced neuroimaging-derived biomarkers, offer unprecedented insights into endothelial health in cSVD. These biomarkers not only aid in early diagnosis but also enable more accurate risk stratification and monitoring of therapeutic responses. Concurrently, this review delves into the latest preclinical and clinical trial progress of innovative therapeutic strategies targeting endothelial repair. By bridging mechanistic insights with clinical translation, this review aims to highlight novel pathways for early intervention and personalized management of cSVD, thereby advancing the field beyond previous reviews that mainly focused on established knowledge. Relevant studies were retrieved from databases such as PubMed and Web of Science, covering the period up to 2025, to synthesize the latest evidence on endothelial dysfunction in cSVD. This review not only synthesizes current knowledge on endothelial dysfunction in cSVD but also critically evaluates the diagnostic and prognostic utility of emerging endothelial biomarkers and discusses recent therapeutic innovations, providing a more forward-looking perspective for researchers and clinicians.

## 1 What is cerebral small vessel disease?

**Figure 1:**
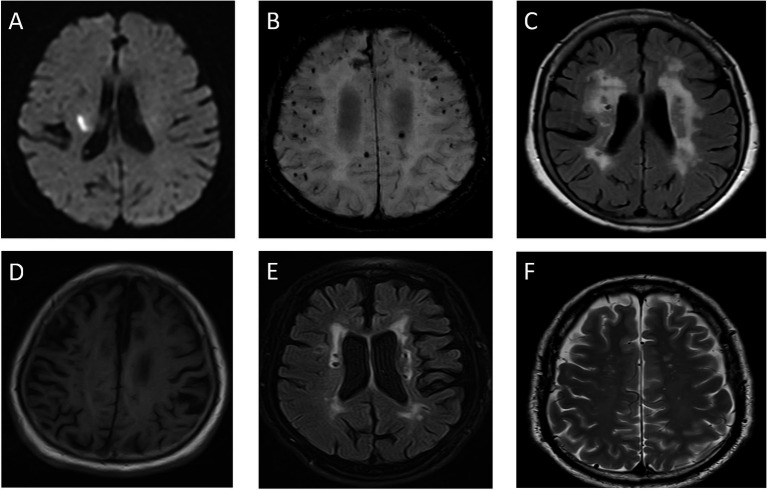
Imaging of cerebral small vessel disease. **(A)** DWI sequence shows hyperintense lesion in right cerebral hemisphere, indicating acute ischemic lesion. **(B)** SWI sequence reveals multiple hypointense cerebral microbleeds (CMBs) in brain parenchyma. **(C)** FLAIR sequence displays hyperintense white matter lesions adjacent to lateral ventricles. **(D)** T1WI shows brain atrophy with widened sulci and enlarged ventricles. **(E)** FLAIR sequence demonstrates small patchy hyperintense lacunar infarction lesions. **(F)** T2WI shows dilated perivascular spaces (VRS) as small round hypointense foci.

cSVD refers to the pathological damage of small blood vessels in the brain, including arterioles, venules, and capillaries (1). It is characterized by MRI features such as lacunar infarcts, white matter hyperintensities (WMH), cerebral microbleeds (CMB), enlarged perivascular spaces, and brain atrophy (2) (Figure 1). Clinically, cSVD commonly presents with lacunar strokes and cognitive impairment, but it is also associated with motor issues, Parkinsonism, balance problems, falls, and behavioral changes like depression, apathy, and personality alterations (3). As a result, cSVD is a significant risk factor for disability and the need for nursing home care.

### 1.1 Epidemiology of cerebral small vessel disease

Epidemiological studies reveal a significant burden of cSVD, particularly in low- and middle-income countries (LMICs). While there is growing recognition of the impact of cSVD on global health, specific data on individual stroke subtypes—such as lacunar stroke—remains relatively limited, especially in these regions (4). A recent review by Lam et al. (5) highlights the high prevalence of imaging features associated with cSVD in LMICs. The study reported that between 20.5% and 58.4% of individuals in community, stroke, and dementia groups exhibited moderate-to-severe white WMH, which is a key radiological marker of cSVD (5). These findings indicate that cSVD is a widespread concern, even in populations with limited healthcare resources (6). This data from LMICs aligns with findings from high-income countries, where WMH and other radiological features of cSVD are similarly common (5). However, the need for more comprehensive studies in LMICs is crucial. A deeper understanding of the global prevalence of cSVD and its regional variations would help in tailoring preventive strategies and health policies to address the burden more effectively. Moreover, certain non-western populations, particularly in Asia, show an even higher burden of cSVD, which is often linked to an increased risk of dementia (7). In these regions, the presence of cSVD lesions appears to have a synergistic effect with Alzheimer’s disease (AD), lowering the threshold for the development of clinically significant dementia (8). This means that individuals with cSVD may experience an accelerated decline in cognitive function when combined with AD pathology, leading to earlier and more severe dementia onset.

### 1.2 Detection methods for cerebral small vessel disease

cSVD are primarily identified through brain MRI, which reveals characteristic features such as WMHs, small subcortical infarcts or lacunes, visible perivascular spaces (PVSs), microbleeds, intracerebral hemorrhage (ICH), and brain atrophy (9). However, conventional MRI may only capture the more obvious lesions, missing subtle changes that could be present in other areas of the brain (10). More advanced imaging techniques like diffusion tensor imaging (DTI) are more sensitive and can detect alterations in diffusion properties in regions that appear normal on traditional MRI scans (11). Clinically, cSVD often presents with stroke due to small subcortical infarcts, motor impairments, balance problems, and cognitive decline, particularly affecting executive function and processing speed (1). Additionally, neuropsychiatric symptoms such as apathy, fatigue, depression, and delirium are increasingly recognized as key manifestations (12). The disease can range from asymptomatic cases, where brain lesions are incidentally found on MRI in individuals over 50, to severe cases leading to disability and dementia (13). The progression of cSVD is typically gradual and silent, with many individuals showing no symptoms until the condition becomes clinically significant. This slow progression is also observed in monogenic forms of cSVD, which are often indistinguishable from the sporadic form of the disease.

### 1.3 Pathogenesis of cerebral small vessel disease

#### 1.3.1 Sporadic cSVD

cSVD represents the most prevalent form, predominantly driven by arteriolosclerosis. This condition is closely associated with aging and vascular risk factors, with hypertension being a key contributor. In age-related non-amyloid cSVD, arteriosclerosis of small vessels plays a pivotal role. Additionally, lacunar infarcts in sporadic cSVD can result from microatheromas at the origin of perforating arteries, a finding supported by previous research and modern high-resolution imaging techniques such as 7 T MRI (10).

#### 1.3.2 Amyloid-angiopathy-related cSVD

Cerebral amyloid angiopathy (CAA) is characterized by the deposition of β-amyloid in the blood vessel walls. CAA primarily affects cortical and leptomeningeal vessels, which leads to lobar intracerebral hemorrhage (ICH) (14). This pathological process also significantly contributes to cognitive decline in the elderly population (15).

#### 1.3.3 Genetic causes

Genetic disorders represent another category of causes for cSVD. Conditions such as cerebral autosomal dominant arteriopathy with subcortical infarcts and leukoencephalopathy (CADASIL) fall into this group. Other less common genetic-related causes include venous collagenosis. Additionally, post-radiation angiopathy can also be associated with cSVD in certain cases, further expanding the genetic and acquired factors contributing to this heterogeneous disease (16).

## 2 The relationship between cerebral small vessel disease and endothelial dysfunction

### 2.1 What is endothelial dysfunction?

Endothelial cells (ECs) dysfunction refers to the impaired functioning of the endothelial cells lining blood vessels, which can have significant implications for vascular health and the organs they serve (17). Under normal conditions, endothelial cells help regulate blood flow, act as a selective barrier, and respond to signals involved in inflammatory processes. In the brain, these cells form the BBB, characterized by tight junctions between the cells that protect the brain from harmful substances while allowing the selective passage of molecules into the brain tissue (18).

When endothelial cells become dysfunctional, they lose these protective functions and may acquire new, harmful characteristics. A hallmark of EC dysfunction is a reduced ability to produce and release nitric oxide, which normally helps dilate blood vessels (19). This results in impaired vascular responses. Although endothelial dysfunction and vascular dysfunction are related, they are distinct processes. Vascular dysfunction can also arise independently of ECs, originating from pericytes or smooth muscle cells. Additionally, endothelial activation, characterized by the upregulation of adhesion molecules like VCAM-1 (20), ICAM-1 (21), and E-selectin (22), often occurs in response to inflammation and is linked to endothelial dysfunction but is considered a separate phenomenon.

### 2.2 How does endothelial dysfunction affect cerebral small vessel disease?

ECs play a crucial role in the brain’s vascular system, extending beyond their function as the inner lining of blood vessels. In the brain, ECs are essential for maintaining the integrity of the BBB, regulating molecular transport, and facilitating processes like blood clotting and neurovascular coupling—matching blood flow to neural activity. They also interact with brain cells on their albuminal side, further influencing brain function. When ECs become dysfunctional, these processes are disrupted, contributing to the development and progression of cSVD, a condition linked to various subtypes based on its cause and pathology (23).

In particular, endothelial dysfunction is increasingly recognized as a key factor in the mechanisms driving parenchymal changes in cSVD. Dysfunctional ECs impair the BBB and neurovascular coupling, leading to changes in white matter (WM). This disruption is thought to occur through altered cellular interactions and secretions from ECs. At the molecular level, EC dysfunction is marked by changes in the expression of various proteins and markers that can serve as indicators of disease progression. Animal models of cSVD have helped identify these molecular signatures, providing insight into how EC dysfunction contributes to WM damage.

## 3 The role of endothelial injury biomarkers in the diagnosis of cerebral small vessel disease

Endothelial dysfunction is a central pathogenic mechanism in cSVD, involving the disruption of the BBB and initiation of inflammatory cascades that damage surrounding brain tissue (24). The cerebral endothelium, together with pericytes and astrocytes, forms the BBB and plays a crucial protective role. When compromised by factors such as hypertension, ischemia, or hypoxia, endothelial cells trigger inflammatory and prothrombotic events that contribute to the characteristic brain changes observed in cSVD (Table 1. Key endothelial injury biomarkers and their roles in cSVD diagnosis).

**Table 1:** The role of endothelial injury biomarkers in the diagnosis of cerebral small vessel disease.

Classification of biomarkers	Specific biomarkers	Mechanism of action	Association with cSVD
Adhesion molecules	Intercellular adhesion molecule-1 (ICAM-1)	Facilitate leukocyte recruitment during inflammatory responses	Elevated in cSVD patients; associated with WMH and lacunar infarcts; diagnostic for silent lacunar infarcts (AUC = 0.81; sensitivity, 74%; specificity, 74%)
	Vascular cell adhesion molecule-1 (VCAM-1)	Facilitate leukocyte recruitment during inflammatory responses	Associated with the presence and progression of WMH and lacunar infarcts, reflecting vascular inflammation and endothelial dysfunction
	P-selectin	Involved in early leukocyte rolling and adhesion	Elevated in patients with lacunar infarcts and WMH, indicating the central role of endothelial-inflammatory interactions in pathogenesis
	E-selectin	Promote firm adhesion of immune cells to endothelium (late inflammatory stage)	Elevated in cSVD patients, similar to P-selectin
Nitric oxide pathway and BBB integrity	Endothelial nitric oxide synthase (eNOS)	Generate NO to maintain vasodilation and tight junctions of the BBB	Reduced activity leads to decreased NO production and increased superoxide radicals, disrupting tight junction proteins, enhancing vascular permeability, and promoting microbleeds
	Tight junction proteins (claudin-5, occludin)	Form physical barriers between endothelial cells to maintain BBB integrity	Reduced expression in cSVD leads to BBB breakdown and harmful molecule infiltration; accompanied by abnormal endothelial cell proliferation and angiogenesis
Matrix metalloproteinases (MMPs)	MMP-2	Degrade tight junction proteins, disrupt BBB, and affect surrounding brain tissue	Associated with WMH and BBB dysfunction; genetic polymorphisms may exacerbate endothelial dysfunction
	MMP-9	Degrade tight junction proteins, disrupt BBB, and affect surrounding brain tissue; degrade myelin basic protein, causing white matter damage	Associated with WMH and BBB dysfunction; genetic polymorphisms may exacerbate endothelial dysfunction; secretes HSP90α to inhibit oligodendrocyte maturation
Coagulation and fibrinolysis markers	von Willebrand factor (vWF)	Reflect endothelial injury and procoagulant state	Elevated in cSVD patients, associated with WMH and lacunar infarcts
	Plasminogen activator inhibitor-1 (PAI-1)	Inhibit fibrinolysis, promoting thrombus formation	Increases the risk of persistent thrombi in cerebral vasculature, involved in cSVD thrombogenesis
	D-dimer	Fibrin degradation product reflecting ongoing clot formation and dissolution	Associated with basal ganglia lacunar infarcts, indicating microthrombosis; predictive value for cSVD progression requires further validation
Angiogenic factors	Vascular endothelial growth factor-A (VEGF-A)	Promote endothelial cell proliferation and angiogenesis	Elevated circulating levels correlate with cerebral microbleeds in Alzheimer’s disease and predict stroke/TIA risk; no direct association with WMH
	Hepatocyte growth factor (HGF)	Enhance VEGF-A-driven angiogenesis and participate in vascular repair	Widely associated with cSVD markers (WMH, lacunes, microbleeds, chronic microinfarcts), potentially representing compensatory repair mechanisms

### 3.1 Adhesion molecules

#### 3.1.1 Intercellular adhesion molecule 1 and vascular cell adhesion molecule 1

Intercellular adhesion molecule 1 (ICAM-1) and vascular cell adhesion molecule 1 (VCAM-1) are glycoproteins expressed on endothelial cells that facilitate leukocyte recruitment during inflammatory responses (25–27). Both molecules are consistently elevated in cSVD patients and strongly associated with WMH and lacunar infarcts (28, 29). ICAM-1 demonstrates particularly robust diagnostic potential, with circulating levels differentiating patients with silent lacunar infarcts from healthy individuals (AUC = 0.81; sensitivity, 74%; specificity, 74%) (30). Longitudinal studies show that rising ICAM-1 levels predict cSVD progression and development of new lacunes in asymptomatic individuals. Similarly, elevated VCAM-1 levels correlate with both the presence and progression of WMH and lacunar infarcts, emphasizing their role in vascular inflammation and endothelial dysfunction.

#### 3.1.2 Selectins (P-selectin and E-selectin)

P-selectin and E-selectin participate in different stages of leukocyte recruitment (31, 32). P-selectin, involved in early leukocyte rolling and adhesion, is elevated in patients with lacunar infarcts and WMH (32). E-selectin facilitates firm adhesion of immune cells to the endothelium during later inflammatory stages and shows similar elevation patterns in cSVD patients, further supporting the central role of endothelial-inflammatory interactions in disease pathogenesis (29).

### 3.2 Nitric oxide pathway and blood–brain barrier integrity

#### 3.2.1 Endothelial NO synthase and NO bioavailability

Reduced endothelial nitric oxide synthase (eNOS) activity represents a key feature of endothelial dysfunction in cSVD (33, 34). Aging and hypertension promote reactive oxygen species (ROS) generation, causing eNOS to shift from producing beneficial NO to generating superoxide radicals. This transition not only impairs vasodilation but also disrupts tight junction proteins through nitrosylation, contributing to increased vascular permeability and the microbleeds characteristic of cSVD (23).

#### 3.2.2 Tight junction proteins: claudin-5 and occludin

Claudin-5 and occludin maintain BBB integrity by forming physical barriers between adjacent endothelial cells (35). In mouse models of cSVD, researchers have found that claudin-5 expression is typically reduced, leading to BBB breakdown and allowing harmful molecules to infiltrate brain tissue (36). This dysfunction is associated with increased endothelial cell proliferation—unusual for mature endothelial cells—and aberrant angiogenesis, potentially representing compensatory mechanisms that paradoxically further disrupt tight junctions (37–39).

### 3.3 Matrix metalloproteinases (MMPs)

MMP-2 and MMP-9 are elevated in cSVD and play dual pathogenic roles (40, 41). These enzymes degrade tight junction proteins (claudin-5 and occludin), contributing to BBB disruption, and also affect surrounding brain tissue (42–44). MMP-9 decreases myelin basic protein levels, contributing to white matter damage, while dysfunctional endothelial cells secrete elevated heat shock protein 90α (HSP90α), which impairs oligodendrocyte maturation (45, 46). Genetic factors, including MMP-2 single nucleotide polymorphisms, may influence enzyme expression and exacerbate endothelial dysfunction (45).

### 3.4 Coagulation and fibrinolysis markers

Elevated vWF levels in cSVD patients indicate heightened procoagulant states and endothelial injury, particularly associated with WMH and lacunar infarcts (47, 48). PAI-1, a potent fibrinolysis inhibitor, contributes to thrombotic risk by inhibiting tissue plasminogen activator, leading to clot persistence in cerebral vasculature (49, 50).

As a fibrin degradation product, elevated D-dimer levels reflect ongoing clot formation and dissolution (51). In cSVD, increased D-dimer is particularly associated with basal ganglia lacunar infarcts, suggesting microthrombotic processes, though its predictive value for cSVD progression requires further validation (52).

### 3.5 Angiogenic factors

VEGF-A promotes endothelial cell proliferation and angiogenesis. Elevated circulating levels correlate with cerebral microbleeds in Alzheimer’s disease patients and predict increased stroke/TIA risk over 10-year periods (53, 54). Notably, VEGF-A shows specific association with microbleeds but not with WMH, suggesting distinct pathogenic pathways within cSVD (55).

HGF enhances VEGF-A-driven angiogenesis and shows broad associations with cSVD markers, including WMH, lacunes, microbleeds, and chronic microinfarcts. Elevated HGF levels in cognitive impairment and Alzheimer’s disease likely represent compensatory vascular repair attempts, though these may be insufficient to counterbalance ongoing injury (56).

## 4 Endothelial dysfunction and its role in the treatment of cSVD

Targeting endothelial dysfunction has emerged as a promising strategy for managing cSVD, with novel therapeutic approaches aiming to restore vascular homeostasis and mitigate brain injury. This section explores both conventional and innovative interventions, highlighting their mechanisms and clinical evidence (Table 2). A summary of therapeutic strategies and their applications in cSVD.

**Table 2:** Endothelial dysfunction and its role in the treatment of cSVD.

Classification of therapeutic strategies	Drug name	Mechanism of action	Clinical application and research evidence
Conventional pharmacological interventions	Aspirin	Inhibit platelet aggregation to reduce microvascular injury	Used for ischemic stroke prevention; reduces recurrent stroke risk by ~30% after acute subcortical infarction
	Antihypertensives	Lower blood pressure to alleviate hemodynamic stress on small vessels and improve endothelial function	Intensive antihypertensive therapy reduces WMH progression more effectively than standard blood pressure control
	Statins (e.g., rosuvastatin)	Regulate lipid levels and improve vascular relaxation by enhancing endothelial NO production, reducing endothelial cell apoptosis	Low-dose rosuvastatin slows WMH progression; some studies (e.g., PODCAST) show limited effects on cognitive decline
Novel endothelial stabilization therapies	Cilostazol	Phosphodiesterase inhibitor promoting endothelial cell survival and stabilizing microvasculature	Reduces cognitive decline and gliovascular damage in animal models; LACI-1/LACI-2 clinical trials evaluating efficacy for cSVD-related cognitive decline
Anti-inflammatory therapies	Minocycline	Inhibit neuroinflammation, protect endothelial cells, and improve white matter integrity	Alleviates endothelial injury and reduces white matter damage in cSVD animal models; requires further clinical validation
	Fingolimod, natalizumab, rituximab	Modulate immune responses to alleviate neuroinflammation	Have shown efficacy in neuroinflammatory diseases and may serve as potential therapies for endothelial protection in cSVD
Stem cell-based therapies	Mesenchymal stem cells (MSCs)	Promote BBB repair, remodel microvasculature, reduce Aβ accumulation, and increase pial microvascular density	Improves cognitive function in animal models by repairing damaged endothelial structures and restoring cerebral blood flow (CBF)
	Oligodendrocyte progenitor cells (OPCs), glial-restricted progenitors (GRPs)	Differentiate into oligodendrocytes to promote white matter remyelination	Repairs structural damage in cSVD-related white matter, improving functional and structural outcomes in the brain

### 4.1 Pharmacologic interventions targeting endothelial dysfunction

Pharmacological strategies aimed at improving endothelial function have shown promise in managing cSVD. One of the primary pharmacological interventions is antiplatelet therapy, which is commonly used in ischemic stroke. The administration of antiplatelet agents such as aspirin has been shown to reduce recurrent stroke risk by about 30% after acute subcortical infarction (57). By reducing platelet aggregation, these drugs may help mitigate microvascular injury, which is a key contributor to endothelial damage in cSVD. However, antiplatelet therapy also carries significant risks, such as an increased risk of bleeding. In some patients, especially those with a history of gastrointestinal ulcers or other bleeding disorders, the use of antiplatelet agents may lead to severe hemorrhagic events, potentially outweighing the benefits (58). Moreover, there is evidence of interindividual variability in the response to antiplatelet drugs, with some patients failing to achieve the expected antiplatelet effect, limiting the universal effectiveness of this treatment approach.

Antihypertensive therapy is another cornerstone of cSVD management. Elevated blood pressure is a significant risk factor for endothelial dysfunction and the progression of WMH. Intensive antihypertensive treatment has been associated with less progression of WMH compared to standard guidelines for blood pressure reduction (59). By reducing systemic vascular resistance, antihypertensive agents alleviate the hemodynamic stress on small vessels, thereby reducing endothelial injury. Nevertheless, overly aggressive antihypertensive treatment can also pose risks. Excessive blood pressure reduction may lead to cerebral hypoperfusion, especially in patients with pre-existing cerebrovascular stenosis, potentially triggering ischemic events (60). Additionally, some antihypertensive medications may have adverse side effects, such as electrolyte imbalances or sexual dysfunction, which can affect patient compliance and the long-term effectiveness of the treatment (61).

Statin therapy, primarily used for lipid-lowering, has also been investigated for its potential benefits in cSVD. Statins, such as low-dose rosuvastatin, have been found to slow WMH progression, suggesting that their effects on endothelial function may extend beyond cholesterol reduction (62). Statins may exert direct beneficial effects on endothelial cells by enhancing endothelial nitric oxide production, which in turn improves vascular relaxation and reduces endothelial cell apoptosis. However, the Prevention of Decline in Cognition after Stroke Trial (PODCAST) showed limited effects of statins and blood pressure management on cognitive decline, indicating that the impact of statin therapy on cSVD-related cognitive outcomes may be more complex than initially thought (63). Moreover, statins are associated with a range of side effects, including muscle pain, liver enzyme elevation, and an increased risk of new-onset diabetes, which may limit their long-term use in some patients. Although these therapies remain a standard approach to preventing further cerebrovascular damage in cSVD patients (64), it is crucial to carefully weigh the benefits and risks for each individual patient.

### 4.2 Novel therapeutic strategies targeting endothelial stabilization

Beyond conventional pharmacological therapies, there is growing interest in novel interventions that specifically target endothelial stabilization and repair. Cilostazol, a phosphodiesterase inhibitor, has shown potential in preclinical studies as a means to enhance endothelial function and reduce cerebrovascular damage in cSVD. By promoting endothelial cell survival and stabilizing the microvasculature, cilostazol has been found to reduce cognitive decline and ameliorate gliovascular damage in animal models of cSVD (65). However, translating preclinical findings to clinical practice presents significant challenges. The results from preclinical studies may not always be replicated in human trials due to differences in pathophysiological mechanisms between animals and humans. Additionally, ongoing clinical trials, such as the Lacunar Intervention (LACI-1 and LACI-2) studies, are investigating the efficacy of cilostazol in treating cSVD-related cognitive decline, but these trials are still in progress. There is a risk that the trials may not meet their primary endpoints, and even if they do, the therapeutic window of cilostazol may be narrow, requiring careful dosing and monitoring. Moreover, potential side effects of cilostazol, such as headache, nausea, and palpitations, may limit patient tolerance and compliance, which need to be thoroughly evaluated in larger-scale clinical trials. These trials may provide further insight into endothelial-targeted therapies for cSVD (66, 67), but it is essential to approach the development of cilostazol-based treatments with caution.

### 4.3 Anti-inflammatory therapies for endothelial protection

Endothelial dysfunction in cSVD is often accompanied by neuroinflammation, which exacerbates vascular injury. Minocycline, an anti-inflammatory agent, has demonstrated potential in alleviating endothelial injury and reducing white matter damage in cSVD models. In preclinical studies, minocycline has been shown to decrease neuroinflammation, improve white matter integrity, and enhance cognitive performance in animal models of cSVD (68). Its ability to modulate immune responses and protect endothelial cells makes it a promising candidate for further exploration in clinical settings. Other anti-inflammatory agents, including fingolimod, natalizumab, and rituximab, have shown efficacy in neuroinflammatory conditions and may be potential therapies for endothelial protection in cSVD (69). However, these agents also carry significant risks. Fingolimod is associated with bradycardia and an increased risk of infections, natalizumab has been linked to progressive multifocal leukoencephalopathy, a rare but serious viral brain infection, and rituximab can cause infusion-related reactions and immunosuppression. These risks highlight the need for careful patient selection, close monitoring, and further research to determine the safety and effectiveness of anti-inflammatory therapies for cSVD.

### 4.4 Stem cell-based approaches for endothelial regeneration

Stem cell therapy, particularly mesenchymal stem cells (MSCs), holds great promise for the treatment of cSVD by directly targeting endothelial injury and improving microvascular function. MSC transplantation has been shown to enhance BBB integrity, remodel the microvasculature, and reduce amyloid-beta (Aβ) accumulation in animal models of cSVD, leading to improved cognitive function (70). Additionally, MSCs have been found to increase the density of the pial microvascular network, suggesting that they may have the ability to restore cerebral blood flow (CBF) in cSVD by repairing damaged endothelial structures (71).

The use of oligodendrocyte progenitor cells (OPCs) and glial-restricted progenitors (GRPs), which have the potential to differentiate into oligodendrocytes and remyelinate damaged white matter, is another promising strategy. These cells could potentially repair the structural damage to white matter in cSVD, improving both functional and structural outcomes in the brain (72, 73). The use of stem cell populations that promote endothelial repair and white matter regeneration represents a novel approach to addressing both the vascular and neurological deficits in cSVD.

## 5 Challenges hindering the clinical translation of endothelial dysfunction research

Despite significant progress in understanding endothelial dysfunction, several methodological limitations, biomarker validation issues, and translational hurdles impede the clinical application of related research findings. A number of studies utilize bioinformatics analyses to discover endothelial injury biomarkers like ICAM-1 and VCAM-1, but the lack of validation in large-scale, multicenter clinical cohorts undermines their reliability. For example, although circulating endothelial microparticles and adhesion molecules seem to be associated with cSVD imaging markers, their diagnostic value has not been established in independent studies, as the area under the curve (AUC) values are typically derived from single-center samples (28, 30). The small cohort sizes, with most studies involving fewer than 200 participants, not only introduce selection bias but also fail to account for ethnic and age-related variability, further restricting clinical translation (5).

Moreover, the use of transcriptomic approaches to identify biomarkers has its own drawbacks. These methods mainly capture transcriptional changes, yet many crucial biomarkers, such as eNOS and selectins, are membrane receptors or secreted proteins. Since the functional activity of these biomarkers, including NO bioavailability or adhesion molecule activation, cannot be accurately determined by mRNA levels alone—considering that reduced eNOS expression may not necessarily correlate with decreased NO production due to post-translational modifications like phosphorylation by AKT—this creates a disconnect between molecular profiling and actual functional significance (33, 34). As a result, inactive biomarkers may be incorrectly deemed clinically relevant (17, 23).

In terms of therapeutic translation, most preclinical therapies focus on single molecules, despite endothelial dysfunction being a complex process involving interconnected pathways. For instance, while MMP-9 inhibition can reduce BBB disruption, the simultaneous upregulation of other proteases like MMP-2 may offset the beneficial effects (44). Additionally, animal models of cSVD, such as those based on chronic cerebral hypoperfusion, often lack human-like comorbidities like hypertension and diabetes, making it difficult to predict clinical responses accurately (70). The failure of statins in large clinical trials, such as PODCAST, clearly demonstrates that targeting isolated pathways may not be sufficient to overcome the intricate endothelial-microglial interactions in humans. These multifaceted challenges collectively pose significant obstacles to translating research on endothelial dysfunction into effective clinical applications (68).

## 6 Conclusion

**Figure 2:**
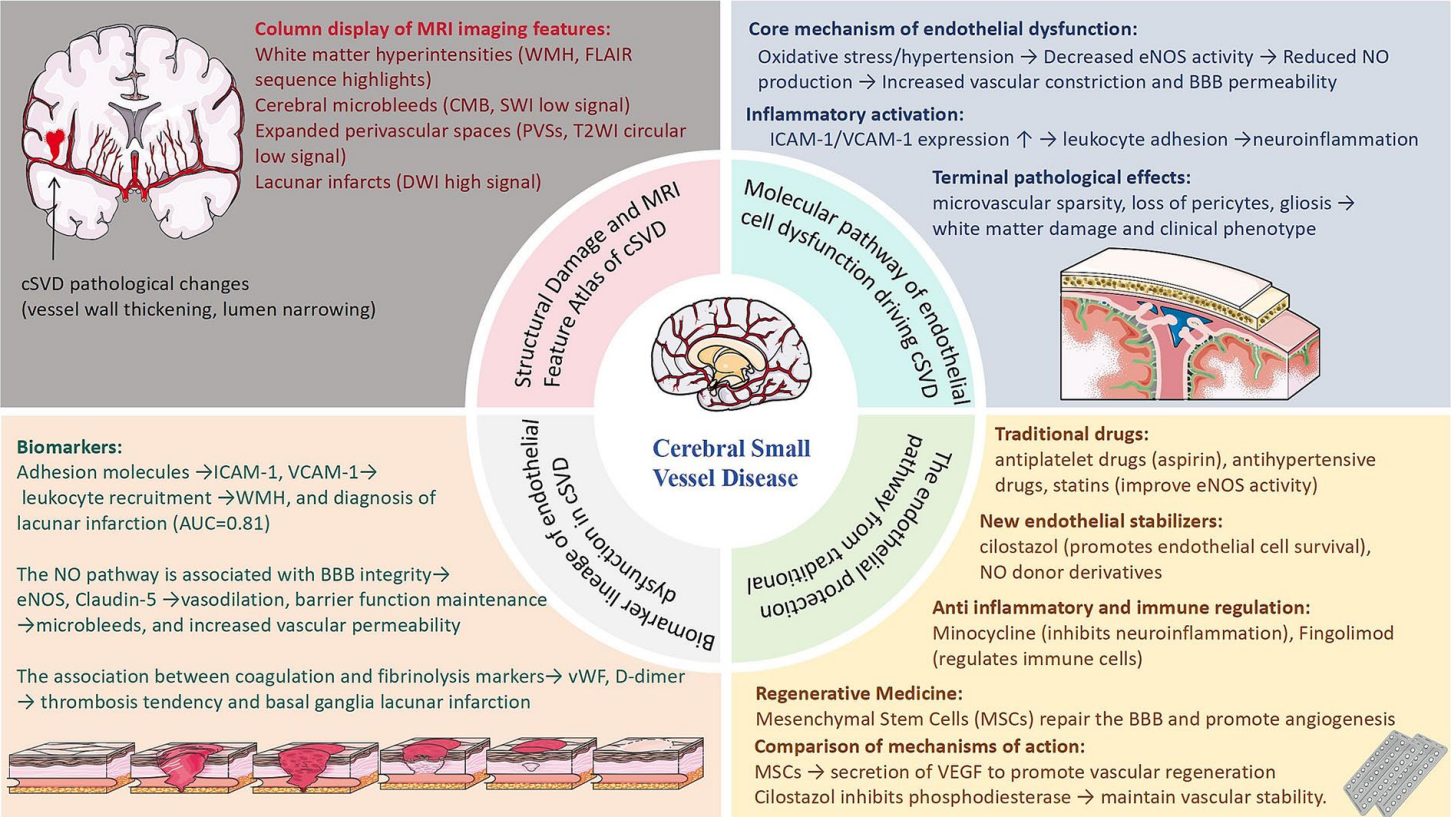
Schematic overview of cerebral small vessel disease (cSVD) pathogenesis, endothelial dysfunction mechanisms, emerging biomarkers, and therapeutic strategies.

Endothelial dysfunction is increasingly recognized as a linchpin in the pathophysiology of cSVD, linking traditional vascular risk factors to end-organ damage in the brain. The identification of endothelial-specific biomarkers, such as soluble thrombomodulin, endothelial progenitor cells, and dynamic contrast-enhanced MRI parameters, holds promise for early diagnosis, risk stratification, and monitoring of therapeutic responses (Figure 2). However, challenges persist in distinguishing endothelial injury from confounding systemic vascular pathologies and standardizing biomarker assays across populations. On the therapeutic front, interventions targeting endothelial resilience—ranging from statins to mesenchymal stem cell therapy—demonstrate potential in preclinical models, yet their efficacy in human cSVD remains to be validated in large-scale trials.

Future research should prioritize multimodal approaches integrating omics technologies, advanced neuroimaging, and deep phenotyping to unravel endothelial heterogeneity in cSVD subtypes. Additionally, addressing the bidirectional crosstalk between endothelial cells, pericytes, and astrocytes may unlock novel therapeutic targets. While endothelial-centric strategies show promise in preclinical and mechanistic studies, their capacity to mitigate the global burden of cSVD-related disability and dementia remains speculative, pending evidence from large-scale interventional trials. As the field progresses, a paradigm shift toward endothelial-focused prevention may be warranted, but this approach requires rigorous validation to establish its clinical utility and impact on public health outcomes.

## Glossary

cSVD: Cerebral small vessel disease
NO: Nitric oxide
WMH: White matter hyperintensities
CMB: Cerebral microbleeds
PVSs: Perivascular spaces
ICH: Intracerebral hemorrhage
DTI: Diffusion tensor imaging
LMICs: Low- and middle-income countries
CAA: Cerebral amyloid angiopathy
ECs: Endothelial cells
WM: White matter
MMPs: Matrix metalloproteinases
eNOS: Endothelial nitric oxide synthase
ICAM-1: Intercellular adhesion molecule 1
AUC: Area under the curve
VCAM-1: Vascular cell adhesion molecule 1
TJ: Tight junction
MBP: Myelin basic protein
HSP90α: Heat shock protein 90α
SNP: Single nucleotide polymorphism
vWF: von Willebrand factor
PAI-1: Plasminogen activator inhibitor type 1
t-PA: Tissue plasminogen activator
VEGF: Vascular endothelial growth factor
TIA: Transient ischemic attack
HGF: Hepatocyte growth factor
CMIs: Chronic microinfarcts
PODCAST: Prevention of Decline in Cognition after Stroke Trial
LACI-1 and LACI-2: Lacunar Intervention Trials 1 and 2
MSCs: Mesenchymal stem cells
CBF: Cerebral blood flow
OPCs: Oligodendrocyte progenitor cells
GRPs: Glial-restricted progenitors
